# Optimization of Eudragit RS100 Nanocapsule Formulation for Encapsulating Perillyl Alcohol and Temozolomide Using Design of Experiments

**DOI:** 10.1021/acsnanoscienceau.4c00057

**Published:** 2025-01-29

**Authors:** Ariane K. Padilha Lorenzett, Tatiane P. Babinski, Vanderlei A. de Lima, Rubiana M. Mainardes

**Affiliations:** † Laboratory of Nanostructured Formulations, Universidade Estadual do Centro-Oeste, Élio Antonio Dalla Vecchia Aveniu, 838, 85040-167 Guarapuava, Paraná, Brazil; ‡ Chemistry Department, Federal Technological University of Paraná UTFPR, Via do Conhecimento, s/n - KM 01 - Fraron, 85503-390 Pato Branco, Paraná, Brazil; § Pharmacy Department, Universidade Estadual do Centro-Oeste, Élio Antonio Dalla Vecchia Aveniu, 838, 85040-167 Guarapuava, Paraná, Brazil

**Keywords:** glioblastoma, temozolomide, perillyl alcohol, intranasal, design of experiment

## Abstract

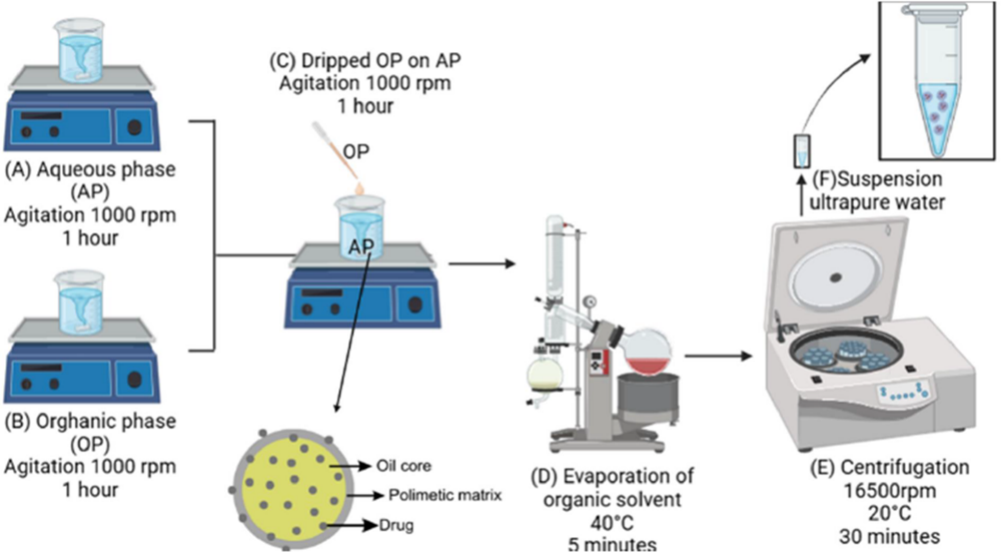

Glioblastoma, an aggressive intracranial tumor, presents significant therapeutic challenges due to the restrictive nature of the blood–brain barrier (BBB), which limits the effectiveness of conventional treatments. This study aimed to develop and optimize a nanoencapsulated system for intranasal delivery of temozolomide (TMZ) and perillyl alcohol (POH), designed to circumvent BBB limitations, utilizing Eudragit RS100 as the encapsulation matrix. A factorial design approach optimized key parameters, including Eudragit RS100 concentration, POH amount, drip rate, and organic-to-aqueous phase ratio. The nanocapsules were characterized by dynamic light scattering, zeta potential analysis, scanning electron microscopy, and high-performance liquid chromatography. The optimized nanocapsules demonstrated a mean diameter of 253 ± 52 nm and a polydispersity index of 0.145 ± 0.037, indicating uniform size distribution. A zeta potential of approximately +20 mV supported colloidal stability. Encapsulation efficiencies were 3.7% for POH and 28.5% for TMZ. This nanoencapsulated delivery system offers a promising approach for glioblastoma treatment, potentially enhancing clinical outcomes and reducing treatment-associated toxicity.

## 1 Background

Glioblastoma is one of the most aggressive brain tumors, characterized by rapid endothelial proliferation, cerebral necrosis, and significant metastatic potential, leading to high mortality rates.^1,2^ Patient survival typically ranges from 8 to 12 months postdiagnosis, depending on tumor location, with better prognoses associated with tumors in more accessible regions like the frontal lobe.^2,3^ Current therapeutic strategies include surgical resection (when feasible), chemotherapy, radiotherapy, and oral antitumor agents.^2^ However, effective treatment is severely limited by the blood–brain barrier (BBB),^4,5^ that not only acts as a physical barrier but also contains active efflux transporters, such as multidrug-resistant proteins (MRP) and P-glycoprotein (P-gp), which pump out substances, including drugs, from the brain into the bloodstream, thereby limiting effective drug concentrations in the target tissue.^6−8^

POH, an amphiphilic monoterpene, crosses biological barriers more readily than TMZ and is thought to exert antitumor effects by modulating gene expression and cellular signaling pathways, including Ras inhibition. Nonetheless, its oral administration often leads to gastrointestinal side effects, which may limit patient adherence.^9−15^

To address these issues, alternative drug delivery strategies are needed. Nanoparticle-based drug delivery systems hold promise due to their capacity for targeted delivery, controlled release, and reduced toxicity. Administering such nanoparticles intranasally offers a potential route to bypass the BBB, utilizing olfactory and trigeminal nerve pathways to deliver drugs directly to the CNS. Intranasal administration of nanoencapsulated drugs could also help mitigate systemic side effects, as it reduces the impact on peripheral circulation and preserves BBB integrity.^16−19^

In this study, we focus on developing nanocapsules using Eudragit RS100 to coencapsulate TMZ and POH for intranasal delivery. These nanostructured systems aim to enhance pharmacokinetic profiles and therapeutic efficacy by directly targeting the CNS. To systematically optimize these nanocapsules, we applied the Design of Experiments (DoE) approach, utilizing multivariate analysis to evaluate and fine-tune key formulation parameters. DoE provides a structured methodology for optimizing nanoparticle formulations by examining the interactions among multiple variables simultaneously. This approach is particularly valuable in nanoparticle formulation, where factors such as polymer concentration, oil ratio, and solvent combinations interact in complex ways that affect particle size, encapsulation efficiency, and zeta potential^20^

One widely used multivariate technique in DoE is Response Surface Methodology (RSM), which enables the exploration of a “design space” by generating models that represent relationships between independent variables and desired outcomes.^20−23^ Specifically, RSM helps in evaluating how adjustments in formulation parameters impact key characteristics of the nanocapsules. These models enable researchers to achieve precise control over formulation characteristics to meet specific therapeutic objectives.^20,21,23^ RSM is further enhanced using desirability functions, which prioritize formulation parameters according to targeted criteria. While univariate analysis can be useful in the initial stages of formulation development, focusing on single-variable effects, it does not capture complex interactions between variables that multivariate approaches like RSM can identify.^24^

The objective of this study is to design and optimize TMZ and POH-loaded nanocapsules intended for intranasal administration as a novel therapeutic approach for glioblastoma. By enabling direct CNS targeting and controlled drug release, these nanocapsules aim to circumvent the BBB, enhance drug bioavailability, and reduce systemic toxicity, thereby improving the therapeutic potential of TMZ and POH for glioblastoma treatment. DoE ensures that the formulation process is efficient and data-driven, optimizing both time and resource allocation and providing a robust foundation for future in vivo studies.

## 2 Experimental Section

### 2.1 Chemicals and Materials

Temozolomide (TMZ), perillyl alcohol (POH), Eudragit RS100, Sorbitan 80, Acetone, Polysorbate 80 Tween 80, Formic acid was purchased from Sigma-Aldrich (St. Louis, USA), Acetonitrile, Deionized and purified water by Milli-Q Reagent Quality Water System (Millipore Corporation, Bedford, MA).

### 2.2 Obtaining Eudragit RS100 Nanocapsules for Encapsulating POH And TMZ

The nanoapsules were prepared using the nanoprecipitation method described by Fessiet al.^25^ First, a solution of 300 mg of Eudragit RS100 in pure acetone was prepared, containing 4 mg of TMZ, 180 mg of POH and 80 μL of Span 80. In another container, an aqueous solution composed of 1% (w/v) tween 80 in ultrapure water was qualified, and the organic and aqueous phase ratio was 1:1. Both solutions were stirred on a magnetic plate for 1 h. Then, the organic phase was gradually added dropwise to the aqueous phase under continuous stirring at 1000 rpm for 1 h to facilitate nanoprecipitation. The organic solvent was subsequently removed using a rotary evaporator at 40 °C for 5 min. The resulting nanocapsule suspension was centrifuged at 16,500 rpm for 30 min at 20 °C. Finally, the nanocapsule pellet was resuspended in 1 mL of ultrapure water and stored at 2–8 °C for further analysis.

### 2.3 Optimization of Obtaining Eudragit RS100 Nanocapsules for Encapsulation of POH and TMZ Using a Design of Experimental (DoE)

To optimize the formulation of Eudragit RS100 nanocapsules for POH and TMZ encapsulation, a factorial design with central points was employed, allowing investigation of the impact of independent variables on key formulation outcomes. The primary goal was to achieve an optimized formulation with favorable properties for drug delivery, focusing on particle diameter, zeta potential, and encapsulation efficiency (%EE). Initially, critical parameters influencing nanocapsule formation via the nanoprecipitation method were identified from literature. A design matrix was generated and randomized using Minitab version 18 software, and statistical analyses, including analysis of variance (ANOVA) and multilinear regression, were applied to assess model significance and variable interactions.

Experiments were systematically randomized to reduce error impact. According with Table 1, the study examined the effects of four independent variables: Eudragit RS100 concentration (X1), POH concentration (X2), drip rate (X3), and the organic-to-aqueous phase ratio (O/W) (X4), on dependent variables such as mean particle diameter (R1), polydispersity index (PDI) (R2), zeta potential (ZP) (R3), and encapsulation efficiency (EE) (R4). A total of 22 experiments were conducted, including six central points to estimate pure error which each point was performed in triplicates. Factor levels were coded and detailed in Table S1. The study’s objectives included obtaining nanocapsules with a mean particle diameter below 250 nm, a narrow size distribution (PDI < 0.2), a mean zeta potential around +20 mV, and encapsulation efficiencies exceeding 30% for TMZ and 10% for POH. The selected targets were based on the literature, which identifies the optimal nanocapsule sizes for intranasal administration, the most suitable polydispersity index to ensure particle homogeneity, the ideal zeta potential for effective interactions with the mucosa, and achievable encapsulation efficiencies for the proposed model.

**Table 1:** Factors, Variables and Levels of the Factorial Design

		levels				
factor	independent variables	–1	0	+1	dependent variables	
X1	eudragit (% m/V)	2.5	5	7.5	R1	average diameter
X2	oil (POH) (% m/V)	1.5	3	4.5	R2	polydispersity index
X3	drop (mL·min^–1^)	1	2	3	R3	zeta potential
X4	O:W ratio (v/v)	1:1	1:2	1:3	R4	encapsulation efficiency

### 2.4 Physicochemical Analysis

#### 2.4.1 Average Diameter

The average diameter of the nanocapsules was determined using Dynamic Light Scattering (DLS). This technique calculates the hydrodynamic radius (rhrh) based on fluctuations in the intensity of scattered light, which correlates with particle size and their Brownian motion behavior.^26^ For measurement, nanocapsule suspensions were diluted in purified water at a 1:200 (v/v) ratio, placed in polyethylene cuvettes, and analyzed at 25 °C with a 660 nm wavelength. Each sample was analyzed over a 3 min period, with the mean diameter and standard deviation calculated from 10 replicates.

#### 2.4.2 Polydispersity Index (PDI)

PDI was assessed by analyzing the autocorrelation function during DLS measurements, which assesses particle size uniformity. The PDI reflects the relative variability in particle size, indicating the degree of homogeneity of the nanocapsule suspension.^27^

#### 2.4.3 Zeta Potential

Zeta potential was measured based on the electrophoretic mobility of the nanocapsules. For this, nanocapsules were resuspended in a 1 mM potassium chloride (KCl) solution at a dilution ratio of 1:100. The suspension was placed in an electrophoretic cell, maintained at 25 °C, and analyzed over a range of ±150 mV. All measurements were conducted in replicates, with results reported as mean values with their standard deviations.

#### 2.4.4 Morphology of Nanocapsules

The morphological characteristics of the nanocapsules were examined using a scanning electron microscope (SEM, TESCAN). For image acquisition, a small volume of the nanocapsule suspension was carefully deposited onto a sample holder. Following the drying process, the sample surface was coated with a thin layer of gold.

#### 2.4.5 Encapsulation Efficiency

The encapsulation efficiency of POH and TMZ within the nanocapsules was determined indirectly using High-Performance Liquid Chromatography with a Diode Array Detector (HPLC/DAD). The chromatographic conditions were based on previously established methods^28^ using a Waters 2695 Alliance High-Efficiency Liquid Chromatograph coupled with a 2998 DAD detector. The mobile phase consisted of acetonitrile (ACN) and water acidified with formic acid (CH_2_O_2_) in a 60:40 (v/v) ratio, utilizing isocratic elution at a flow rate of 1 mL/min, and a column temperature of 20 °C. Detection wavelengths were set at 193 nm for POH and 328 nm for TMZ, with separation achieved on an Eclipse C18-XDB reversed-phase column. To assess encapsulation efficiency, the supernatant obtained after ultracentrifugation during the final stage of nanocapsule preparation was carefully collected, diluted 1:10 in the mobile phase, filtered through a 0.22 μm membrane, and then injected into the HPLC system. The ultracentrifugation process separated the nanocapsules into a pellet, with the free drug remaining in the supernatant, allowing for indirect quantification of the unencapsulated drug concentration.

To ensure accuracy, the filtration step removed any remaining particles that might interfere with the analysis. This filtration process maintains the integrity of the measurement by ensuring that any unencapsulated drug in the supernatant is accurately quantified, while encapsulated drug remains in the nanocapsules. The encapsulation efficiency was then calculated based on the concentration of free drugs in the supernatant, using eqs 1 and 2 to indirectly determine the percentages of POH and TMZ successfully incorporated into the nanocapsules:
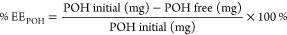
1
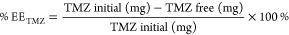
2where initial POH/TMZ represent the initial concentrations of POH and TMZ added during nanocapsule preparation, and free POH/TMZ indicate the concentrations of unincorporated drug remaining in the supernatant after nanocapsule formation. Encapsulation efficiency (EE%) is reported as the mean and standard deviation from replicate analyses.

## 3 Results and Discussion

### 3.1 Optimization of the Preparation of TMZ and POH Nanocapsules by Nanoprecipitation Using a 2^4^ Factorial Design

A total of 22 experimental trials in triplicate were performed to assess the influence of various process variables, (factors) on the response variables: average diameter (R1), polydispersity index (PDI—R2), zeta potential (ZP—R3), and encapsulation efficiency for TMZ (%EE—R41) and POH (%EE—R42). Among these, six trials were designed as central points, representing identical concentrations repeated to assess pure regression error, referred to as F3. Detailed results of each experiment trial are presented in Table S2.

After completing all trials and collecting results, each dependent variable was individually analyzed to examine the relationship between factor levels and outcomes. Multiple regression techniques, ANOVA, *F*-value calculations (based on the Fisher distribution), and *p*-value determination were performed using Statistics software to validate the model’s ability to represent the data accurately and to determine the quality of fit for each response variable. It is possible to conduct an optimization test using the global response of all parameters to optimize the nanocapsule based on all dependent variables. However, this approach may not effectively represent individual parameters due to potential variations that might not be significant for each variable in this model. For this reason, each variable was analyzed separately. Subsequently, a desirability test was performed to integrate the information and ultimately optimize the nanocapsule.

The *F*-test was employed to assess the variance differences between data groups, with the null hypothesis assuming no significant variance differences between groups.^22^ This approach allowed us to determine the influence of specific independent variables (such as pH, polymer concentration, and drip rate time) on the dependent outcomes, including particle size and zeta potential.

Each *p*-value calculated represented the probability that observed results would occur under the null hypothesis, which assumes no effect of the factors tested.^22^ A *p*-value below 0.05 was considered statistically significant, indicating that the corresponding factor had a meaningful effect on the properties of the nanoparticles.

To ensure clarity, the effects of each factor are discussed individually in the following sections.

#### 3.1.1 Particle Size

Mathematical models were applied to identify the main factors influencing the formation of nanocapsules. For the average diameter, a linear model with interactions was obtained (eq 3):
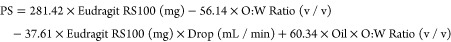
3

The coefficient of determination (*R*^2^) measures the proportion of variability in the dependent variable explained by the model. For particle size, the *R*^2^ value was 85.52%, indicating that the model successfully accounts for most of the data variation, providing an adequate fit. High *R*^2^ values close to 1 reflect strong explanatory power, suggesting that the model captures critical variables influencing nanoparticle characteristics. However, it is essential to monitor for potential overfitting, especially in complex models with numerous variables or interactions, which could artificially inflate *R*^2^. In the context of nanoparticle formulation, high *R*^2^ values are desirable, as they indicate the model’s reliability in predicting crucial properties such as particle size and zeta potential, both vital for stability and therapeutic effectiveness.^29^

According to the ANOVA results, the linear model with interactions effectively captures the complexities of this system. This is supported by the calculated *F*-value for the regression (5.52), which exceeds the critical *F*-value (3.14), indicating that the model is statistically significant at an α = 0.05 level. The p-values reveal the statistical relevance of the model’s factors, with Eudragit concentration (*p* = 0.038) showing a direct and significant impact on particle diameter. Other critical interactions include the organic-to-aqueous phase ratio (O/W) (*p* = 0.0035) and its interaction with oil concentration (*p* = 0.002), both of which significantly affect the outcome, emphasizing the necessity of considering both main effects and interactions in optimizing nanocapsule formulations for specific therapeutic applications, such as intranasal delivery for glioblastoma treatment.

**Figure 1:**
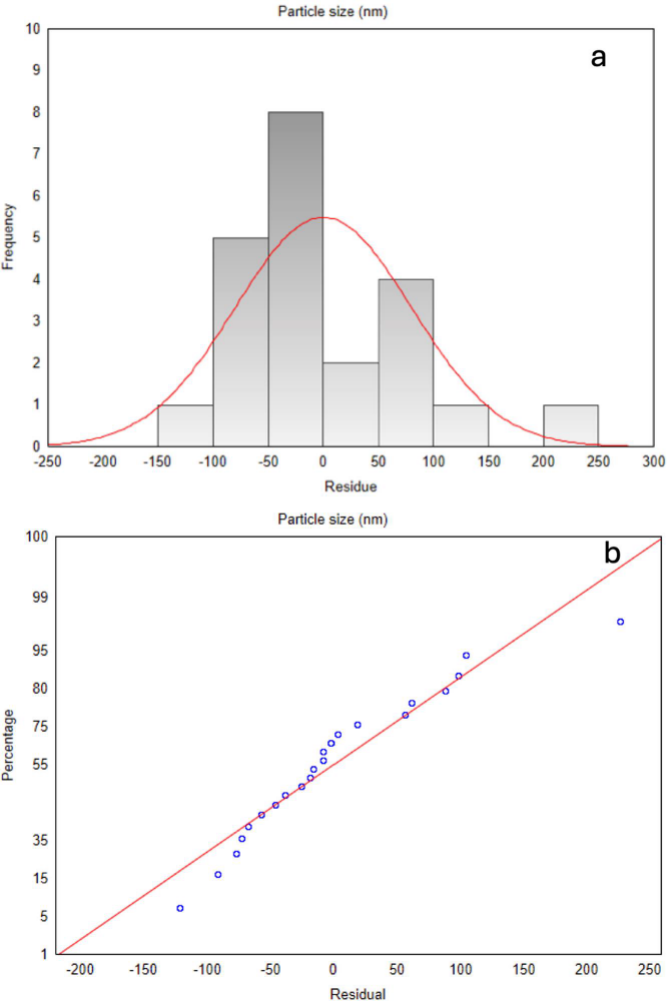
(a) Histogram of particle size residuals (b) particle size normal probability residual plot.

Residual plots were generated to further evaluate the model’s fit quality, as shown in Figure 1. In the histogram (Figure 1a), residuals are symmetrically distributed around zero, with a minor concentration of negative values. The overlaid red curve represents a normal distribution, indicating that the residuals approximate normality. The normal probability plot of residuals (Figure 1b) also shows that most points align with the normality line, with minimal deviations at the ends, which is typical in practical analyses.

An adequate model should exhibit randomly distributed residuals around zero, confirming that it accurately captures the underlying data trends. Random, homoscedastic residuals (i.e., constant variance) support the model’s suitability, while larger or frequent residuals would suggest inaccuracies, potentially impacting the model’s reliability and the validity of its conclusions. This aspect is particularly crucial in nanoparticle research, as small variations in parameters can profoundly impact formulation stability and therapeutic efficacy.^29^

Assessing both *R*^2^ and residuals offers a comprehensive understanding of model quality. While *R*^2^ indicates the overall fit, residual analysis provides insights into the model’s accuracy and unbiased nature. A high *R*^2^, combined with low-magnitude, randomly distributed residuals, enhances confidence in the model’s predictive capability.^29^ confirming that the linear model is robust for predicting particle size in this formulation context.

**Figure 2:**
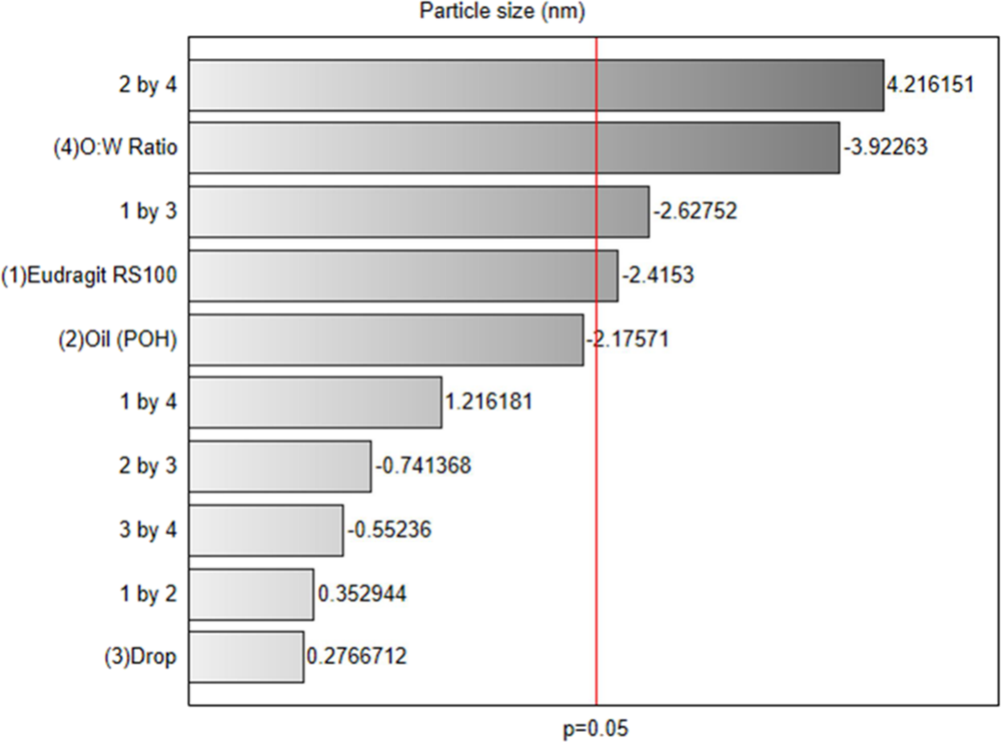
Pareto chart of particle size (*p* < 0.05).

The Pareto chart (Figure 2) from the factorial design analysis illustrates the influence of various factors on the mean particle diameter, a critical parameter in nanocapsule formulation. The red reference line at *p* = 0.05 in Figure 2 denotes the threshold for statistical significance. Notably, only the primary effect of the O/W and the interaction between oil concentration and O/W ratio were statistically significant at the 5% level. Factors with minor effects, such as drip rate, showed no statistical significance and were deemed secondary in optimizing mean particle diameter.

Among the evaluated factors, the interaction between oil concentration and O/W ratio was the most significant, showing a positive effect on particle diameter. Specifically, increasing both oil concentration and O/W ratio resulted in larger particle sizes. While this finding is relevant, it may pose a limitation for intranasal administration, as particle sizes exceeding 250 nm are less effective for crossing biological membranes and are more prone to phagocytosis.^30^ Adjustments to oil concentration and O/W ratio were therefore made to produce nanocapsules within the desired particle size range.

Conversely, the O/W ratio alone had a notable negative effect on particle diameter, with higher O/W ratios leading to a substantial decrease in particle size. Other factors, such as the interaction between Eudragit concentration and drip rate, as well as oil concentration and Eudragit, exhibited adverse effects, though less pronounced. These results indicate that to minimize particle size, particular attention should be given to fine-tuning these variables.

**Figure 3:**
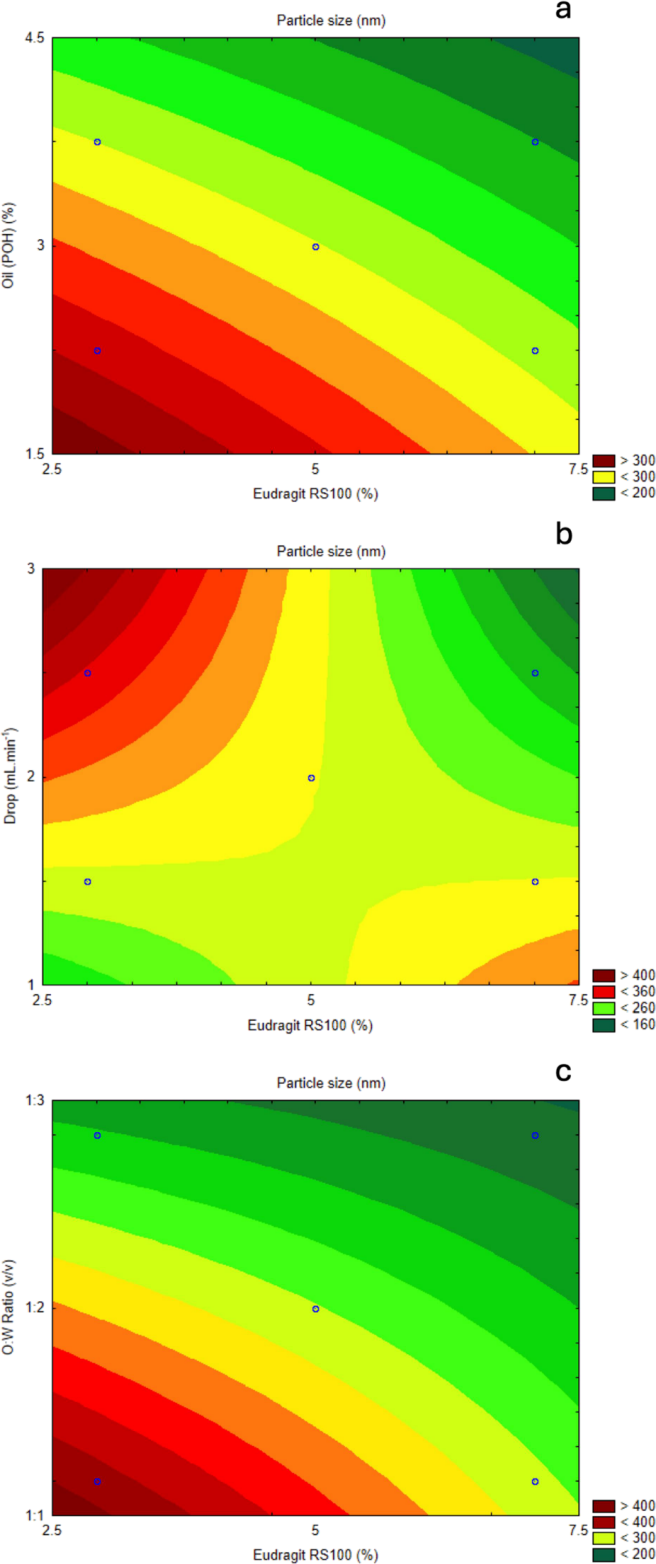
(a) Contour plot of response surface between Eudragit RS100 (%) and oil concentration (%) related to particle size. (b) Contour plot of response surface between Eudragit RS100 (%) and drip rate (mL·min^–1^) related to particle size. (c) Contour plot of response surface between Eudragit RS100 (%) and O:W ratio (v/v) related to particle size.

Contour plots illustrate the interaction between key factors influencing particle size. The contour plot of Eudragit concentration and O/W ratio (Figure 3c) shows that reducing the O/W ratio tends to increase the average particle diameter, especially at lower Eudragit levels. As Eudragit concentration rises, the O/W ratio’s effect on particle size diminishes, potentially due to stabilization effects linked to increased zeta potential at higher Eudragit concentrations. Elevated zeta potential results in stronger electrostatic repulsion between particles, reducing coacervation and aggregation, thereby maintaining particle stability and accurate size measurements through DLS.^31,32^ This trend aligns with the Pareto chart, further underscoring the O/W ratio as inversely proportional to particle diameter.

When examining the effects of POH concentration combined with Eudragit percentage (Figure 3a), higher oil (POH) concentrations were found to reduce particle diameter, particularly at lower Eudragit levels. As Eudragit concentration increased, the impact of oil concentration on particle diameter diminished, highlighting a modifiable interaction for achieving the desired particle size. This finding emphasizes the importance of controlling oil content within the nanocapsule formulation to maintain the particle size within optimal limits.

Finally, the interaction between drip rate and Eudragit concentration was also investigated (Figure 3b). Drip rate, a standardized process for nanocapsule preparation, involves precisely controlling each drop of the organic phase as it is added to the aqueous phase. Results indicated that lower drip rates and lower Eudragit concentrations led to larger particle diameters. However, increasing the drip rate decreased particle size, particularly at lower Eudragit levels, suggesting that drip rate is a potentially critical factor for precise particle size control, even though it was not statistically significant in this model. Therefore, while drip rate should be monitored, it may not be a primary factor in nanocapsule size optimization.

#### 3.1.2 Polydispersity Index (PDI)

A linear model with interactions (eq 4) was developed for analyzing the PDI, representing particle size uniformity in the nanocapsule formation.
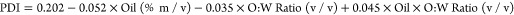
4

The coefficient of determination (*R*^2^) was 90.56%, suggesting that the model is robust in explaining the variability within the data set. ANOVA results further indicated that oil concentration and the O/W ratio significantly influence particle size uniformity. The calculated *F*-value for regression (8.63) exceeded the critical *F*-value of 3.14 at a 0.05 significance level, affirming that the model accurately represents the data set. These findings show that adjustments in these factors are essential for controlling PDI, as both oil concentration and the O/W ratio had substantial effects on particle size distribution.

Additionally, the interaction between oil concentration and O/W ratio was statistically significant (*p* < 0.05), underscoring the need to account for both main effects and their interactions when optimizing nanocapsule formulations for a more uniform particle size distribution. Variables such as Eudragit concentration and drip rate showed no statistical significance, suggesting a lesser role in influencing PDI and allowing them to be adjusted as secondary factors.

**Figure 4:**
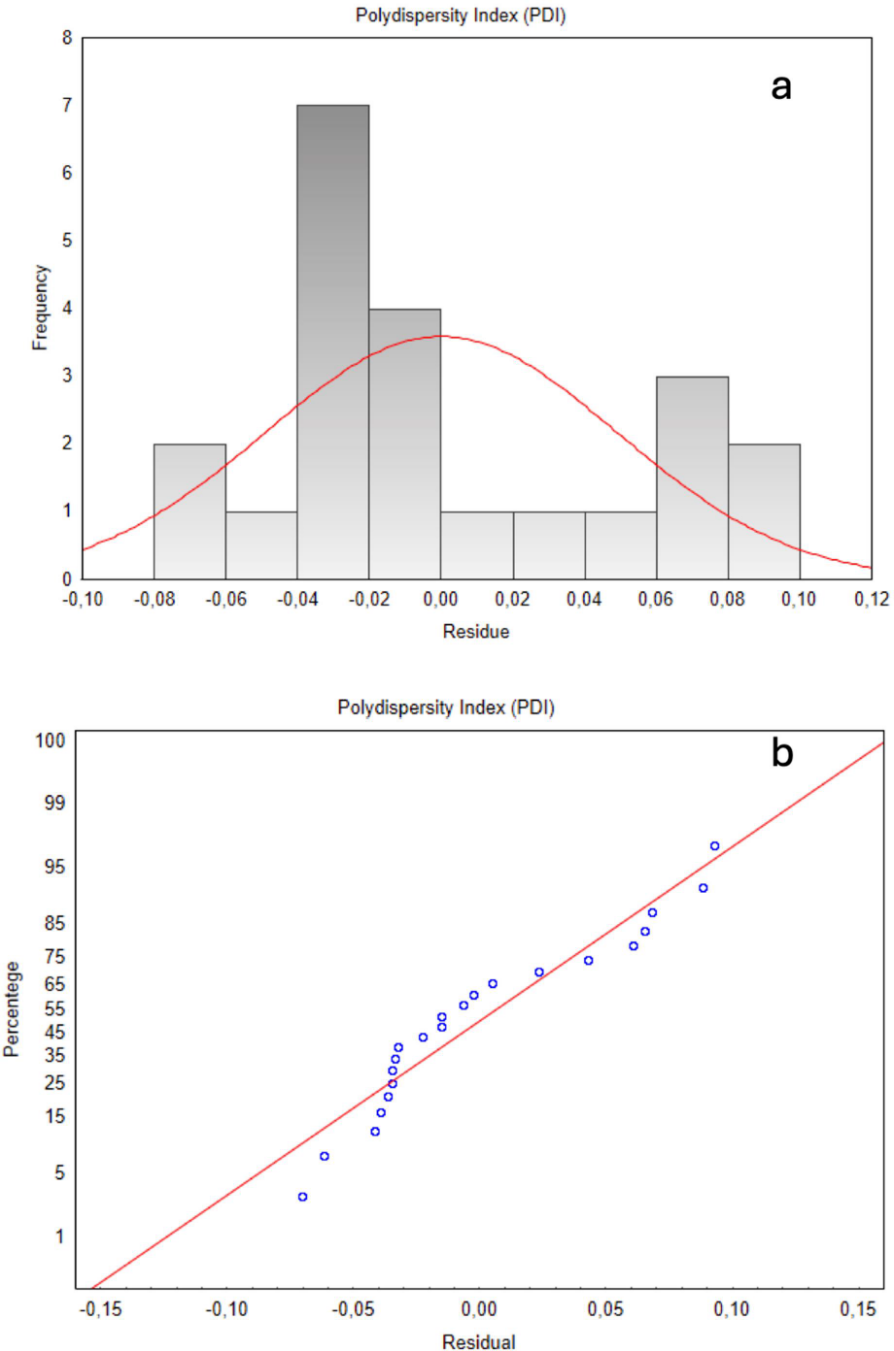
(a) Histogram of PDI residuals (b) PDI normal probability residual plot.

Residual analysis (Figure 4) provided further validation for the model. The histogram (Figure 4a) of PDI residuals shows a concentration around zero with a slight left skew, indicating that while the model is largely unbiased, minor asymmetries exist. The approximation to a normal distribution curve suggests that residuals are near normal, supporting model adequacy. In the normal probability plot (Figure 4b), most points align closely with the normality line, with only minor deviations at the extremes, which do not compromise the model’s validity.

**Figure 5:**
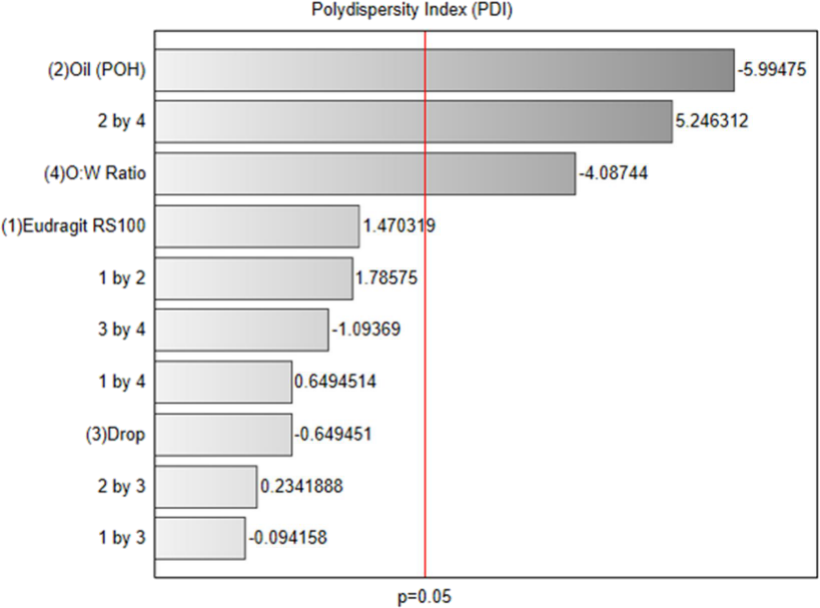
Pareto chart of PDI (*p* < 0.05).

The Pareto chart (Figure 5) illustrates the main factors affecting particle size uniformity. The analysis identified oil concentration (POH) as the most significant factor, with a negative impact of −5.99 on PDI, suggesting that increasing oil quantity notably reduces polydispersity and promotes uniform particle size distribution. The interaction between oil and O/W ratio had a negative effect of −5.25, emphasizing the importance of this combination in minimizing PDI. Similarly, the O/W ratio itself had a significant negative impact of −4.09, reinforcing that optimizing this ratio improves particle uniformity. Meanwhile, factors such as Eudragit concentration and drip rate exhibited lesser impacts on PDI, falling below the significance threshold, and are thus considered secondary in this formulation’s optimization.

**Figure 6:**
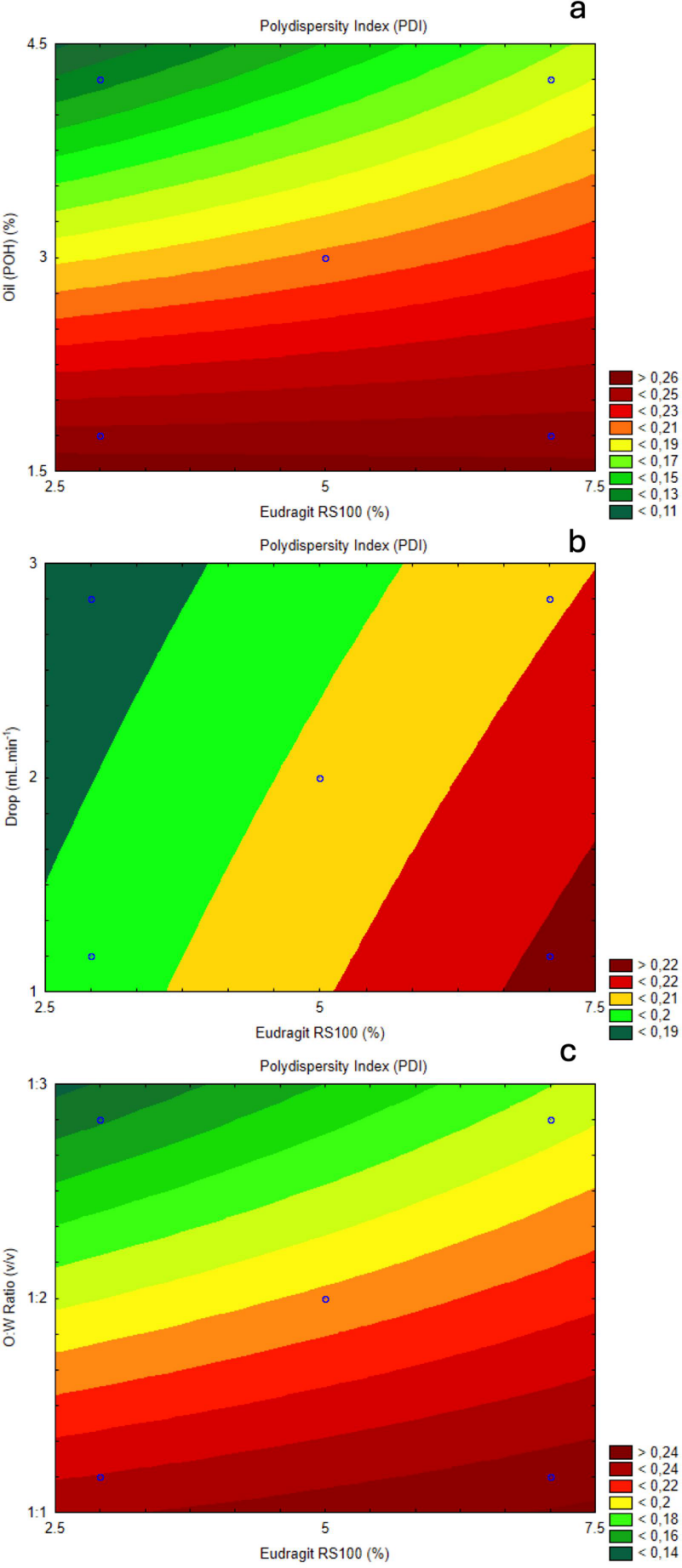
(a) Contour plot of response surface between Eudragit RS100 (%) and oil concentration (%) related to PDI. (b) Contour plot of response surface between Eudragit RS100 (%) and drip rate (mL·min^–1^) related to PDI. (c) Contour plot of response surface between Eudragit RS100 (%) and O:W ratio (v/v) related to PDI.

Contour plots were generated to further examine the impact of oil concentration (POH) and Eudragit concentration on PDI (Figure 6a). The plot reveals that PDI increases as oil concentration decreases, particularly when Eudragit levels are high. This trend indicates the importance of maintaining higher oil concentrations to minimize polydispersity, especially in formulations with increased Eudragit, thereby enhancing particle uniformity. Other contour plots highlight the effects of drip rate and Eudragit concentration on PDI (Figure 6b). The analysis showed that higher drip rates combined with lower Eudragit concentrations resulted in lower PDI values, indicating more uniform particle formation. Conversely, lower drip rates and higher Eudragit levels led to increased PDI, suggesting that adjustments in these parameters are necessary to achieve nanocapsules with desired size distributions.

Lastly, the interaction between O/W ratio and Eudragit concentration (%) was assessed for its effect on PDI (Figure 6c). Increasing the O/W ratio was found to increase PDI, especially at higher Eudragit levels. The gradient from green to red in the contour plot highlights that careful control of the O/W ratio is crucial for achieving uniform particle sizes, particularly with intermediate Eudragit concentrations. These findings underscore the importance of a balanced formulation to ensure homogeneity in nanocapsules, which is essential for their therapeutic efficacy.

#### 3.1.3 Zeta Potential

As with other parameters, the zeta potential exhibited a linear model with interactions, represented by the regression equation


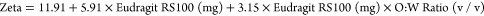
5

**Figure 7:**
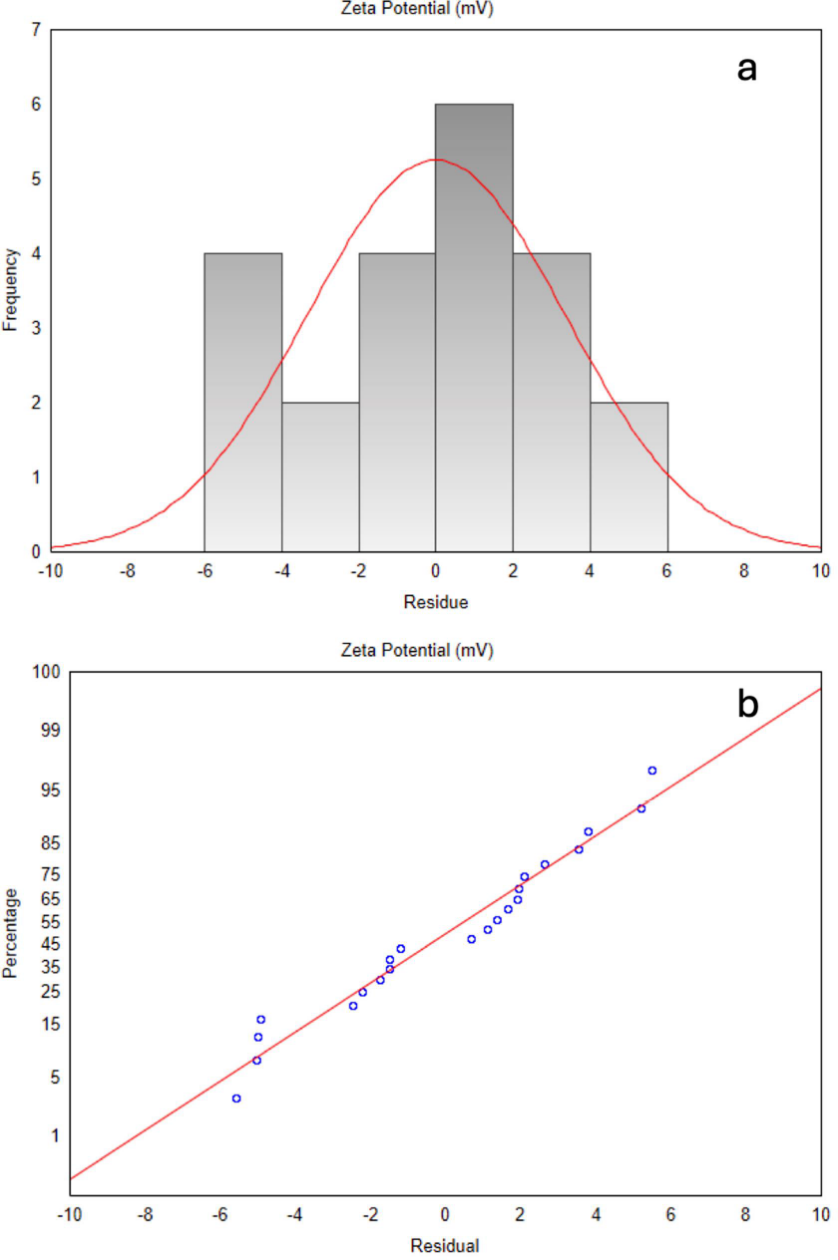
(a) Histogram of zeta potential residuals (b) zeta potential normal probability residual plot.

The coefficient of determination (*R*^2^) for zeta potential was 80.37%, indicating that the model provides an adequate fit for this data set. ANOVA results highlighted Eudragit concentration as the most influential factor, with a *p*-value of 0.001, significantly below the 0.05 threshold, confirming its critical role in increasing zeta potential due to its cationic nature, which enhances nanocapsule stability.^33^ Additionally, the interaction between Eudragit concentration and the O/W ratio was statistically significant (*p* = 0.031), underscoring its importance in formulation optimization for improved colloidal stability. Other factors did not show significant effects on zeta potential, suggesting minimal impact. The model’s suitability was further confirmed by the calculated *F*-value (3.69), which slightly exceeded the tabulated *F*-value (3.14), indicating that the model effectively explains the zeta potential variation observed. Residual analysis (Figure 7a) supports model validity, as residuals are symmetrically distributed around zero, and the histogram closely approximates a normal distribution. The normal probability plot (Figure 7b) shows most points aligning with the normality line, with minor deviations at the tails, which are expected in practical data and do not compromise the model’s integrity.

**Figure 8:**
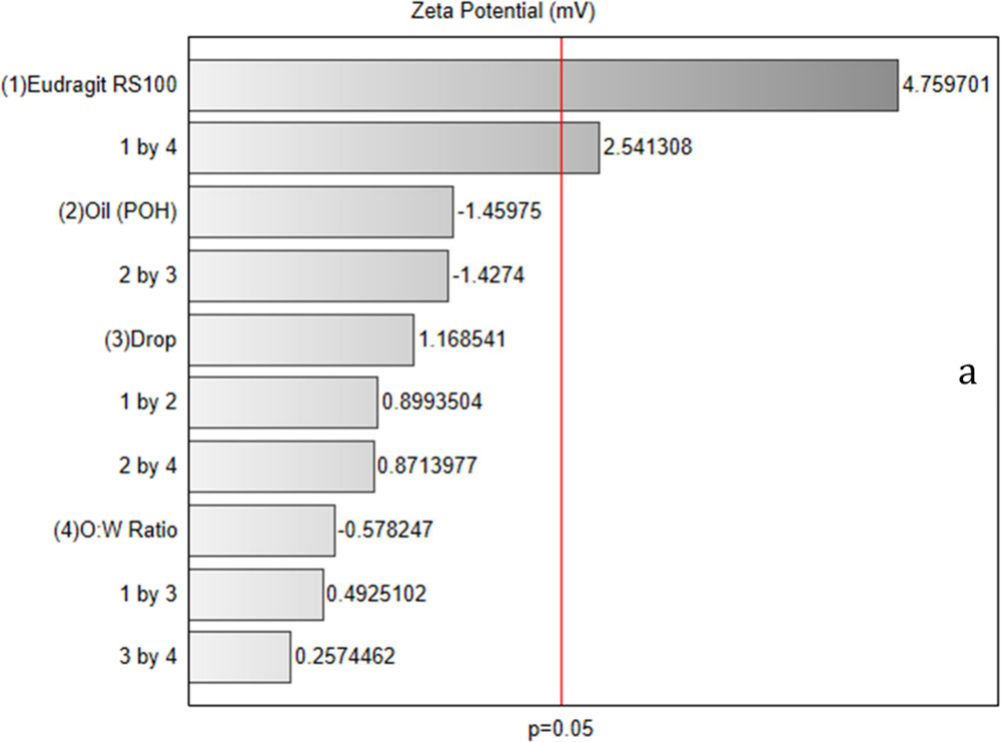
Pareto chart of zeta potential (*p* < 0.05).

The Pareto chart (Figure 8) revealed that Eudragit concentration had the most substantial positive effect on zeta potential. Higher concentrations of Eudragit RS100 increased zeta potential, which contributes to enhanced colloidal stability by providing stronger electrostatic repulsion between particles, thereby reducing aggregation tendencies.^34^ The interaction between Eudragit concentration and the O/W ratio also had a significant positive effect, indicating the necessity of optimizing these factors to improve suspension stability. In contrast, factors such as oil concentration and drip rate showed minor, nonsignificant impacts on zeta potential, suggesting they can be adjusted as secondary considerations.

**Figure 9:**
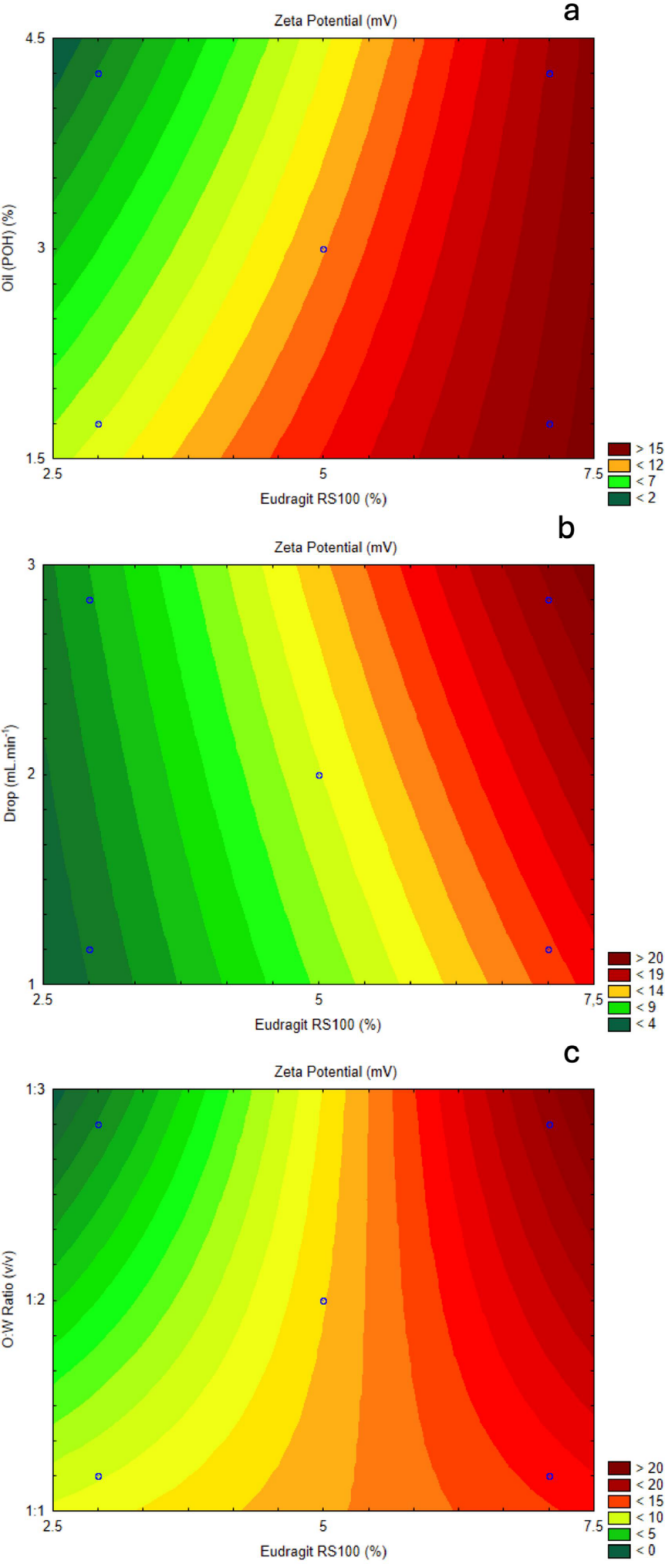
(a) Contour plot of response surface between Eudragit RS100 (%) and oil concentration (%) related to zeta potential. (b) Contour plot of response surface between Eudragit RS100 (%) and drip rate (mL·min^–1^) related to zeta potential. (c) Contour plot of response surface between Eudragit RS100 (%) and O:W ratio (v/v) related to zeta potential.

A contour plot analysis further explored how zeta potential varies as a function of two independent variables. The contour plot for Eudragit RS100 concentration and oil concentration (Figure 9a) demonstrates that higher Eudragit concentrations consistently lead to increased zeta potential, with minimal influence from oil concentration. The color shift from green to red indicates that higher Eudragit levels maximize zeta potential, emphasizing its role in stabilizing colloidal suspensions. The effects of drip rate and Eudragit RS100 concentration on zeta potential were also examined (Figure 9b). Increasing Eudragit RS100 concentration leads to significant increases in zeta potential regardless of drip rate. However, the combination of higher drip rates with high Eudragit concentrations results in the highest zeta potential values, suggesting that maintaining higher drip rates could help enhance colloidal stability by maximizing Eudragit RS100s impact, although this factor did not reach significance in the Pareto analysis.

Finally, the interaction between Eudragit RS100 concentration and O/W ratio was assessed (Figure 9c). The plot shows that a lower O/W ratio combined with high Eudragit concentrations yields the highest zeta potential values. The red and orange regions indicate the most favorable combinations for maximizing zeta potential, once again emphasizing the dominant role of Eudragit RS100 in achieving stable nanocapsule formulations.

#### 3.1.4 Encapsulation Efficiency

The ANOVA for encapsulation efficiencies of POH and TMZ indicated that the linear models applied were not statistically significant in explaining the variations observed in these parameters. This is reflected in the low coefficients of determination (*R*^2^ = 34.25% for TMZ and *R*^2^ = 54.78% for POH), suggesting that the models explained only a limited portion of the variability in encapsulation efficiency for both compounds. Due to the low explanatory power, surface plots or Pareto charts for these variables were excluded from the article. The lack of model significance suggests that the formulation conditions examined (treatments) exerted a relatively uniform influence on the encapsulation efficiencies of POH and TMZ, without any distinct impact from individual factors.

The DoE-derived models described in this study were structured to examine changes in the properties of the nanocapsules univariately with respect to the input variables. In other words, the analysis focused on how each independent parameter affected the selected dependent variable. This approach enables a detailed assessment of how individual variables influence specific properties, such as mean diameter, polydispersity index, zeta potential, and encapsulation efficiency. However, it is important to emphasize that multivariate models were also considered and applied, aiming to comprehensively optimize the key properties of the NPs. Notably, the desirability function at the final stage incorporates all parameters collectively to optimize the final formulation based on the mathematical models.

#### 3.1.5 Hierarchical Clustering (HCA), and Principal Component Analysis (PCA)

**Figure 10:**
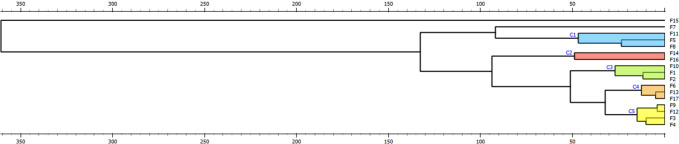
Hierarchical Clustering Dendrogram of Nanocapsule Formulations Based on the dependent variables.

The dendrogram produced from hierarchical clustering analysis (HCA) illustrates the similarity relationships between nanoparticle formulations (F1, F2, F3, etc.), based on their distances or dissimilarities (Figure 10). The horizontal axis at the top represents the dissimilarity scale, with greater branch distances indicating higher dissimilarity among formulations. Each cluster groups formulations with similar characteristics, potentially indicating shared properties. The dendrogram identifies five main clusters (C1, C2, C3, C4, and C5), distinguished by color: C1 (blue): contains formulations F5, F8, and F11. C2 (red): includes formulations F14 and F16. Clusters C1 and C2 exhibit the greatest heterogeneity, as indicated by their longer horizontal bars, suggesting significant differences within these groups. Conversely, C3 (green), comprising F1, F2, and F10, and C4 (orange), including F6, F13, and F17, show higher internal similarity, with low dissimilarity levels. C5 (yellow) contains F3, F4, F9, and F12, where F9 and F12 (C5) and F13 and F17 (C4) show particularly high homogeneity. Formulations F7 and F15 did not cluster, suggesting they are significantly different, possibly representing unique properties or outliers. In the context of nanoparticle formulations, clusters may indicate distinct formulation classes or groups with similar physicochemical profiles, providing a basis for categorization and further analysis.

The dendrogram aids in improving the quality of the PCA, a statistical tool for visualizing correlations between response variables, which reduces dimensionality while retaining essential variance patterns. PCA transforms original variables into uncorrelated components, simplifying the interpretation of relationships within the data set.

**Figure 11:**
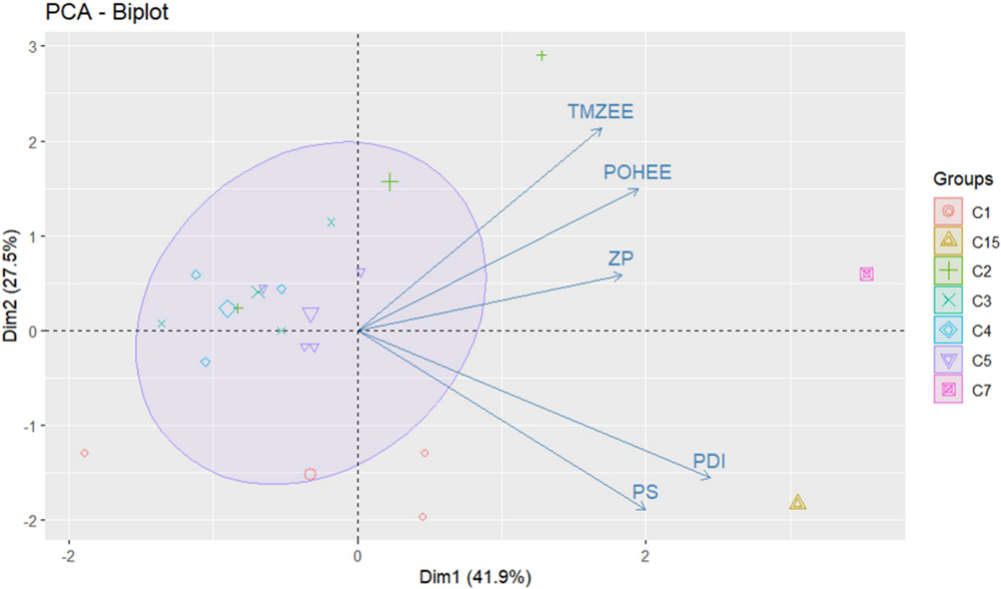
Projection of the dependent variables (loadings) and formulations (scores), illustrating the interrelationships between them.

The PCA plot (Figure 11) shows variations among nanocapsules in terms of encapsulation characteristics, such as TMZ encapsulation efficiency (TMZEE), POH encapsulation efficiency (POHEE), zeta potential (ZP), polydispersity index (PDI), and particle size (PS). This visualization clarifies correlations among variables and provides insights into how different formulations behave based on their physicochemical properties. PCA facilitates understanding of encapsulation performance and nanocapsule stability.

In PCA, principal components are derived from the spectral decomposition of the correlation or covariance matrix. This decomposition yields eigenvalues, representing the variance explained by each component, and eigenvectors, indicating the contribution of each original variable.^35^ Here, PS and PDI are highly correlated, with a positive relationship suggesting that increases in PS correspond to increases in PDI. Similarly, TMZEE, POHEE, and ZP are positively correlated, indicating that increases in TMZEE correspond to increases in POHEE and ZP.

The principal components (Dim1 and Dim2) are linear combinations of original variables explaining most data set variability. Dim1 (horizontal axis) captures the largest data variance, while Dim2 (vertical axis) captures the second largest.^30^ Dim1 is strongly influenced by PS and PDI (Quadrant I), with formulations F7 and F15 positioned farthest to the right due to their high PDI and particle sizes, indicating they are highly correlated with PDI. Formulations F14 and F7 also displayed the highest values for TMZEE and POHEE. In this study, Dim1 and Dim2 together explain 69.4% of total variance (41.9% for Dim1 and 27.5% for Dim2).

Other clusters, including C1 (F5, F8, F11), C3 (F1, F2, F10), C4 (F6, F13, F17), and C5 (F3, F4, F9, F12), presented the lowest values across all nanocapsule characteristics. Nanocapsules in these formulations displayed the smallest particle sizes, lowest PDI values, lowest zeta potentials, and the lowest Eudragit recovery yields.

This analysis is crucial for identifying formulation patterns and optimizing nanocapsule stability and effectiveness. By understanding how different experimental factors interact to influence nanocapsule properties, researchers can make informed adjustments to enhance development efficiency and ensure formulations meet therapeutic needs.

#### 3.1.6 Response Optimization

**Figure 12:**
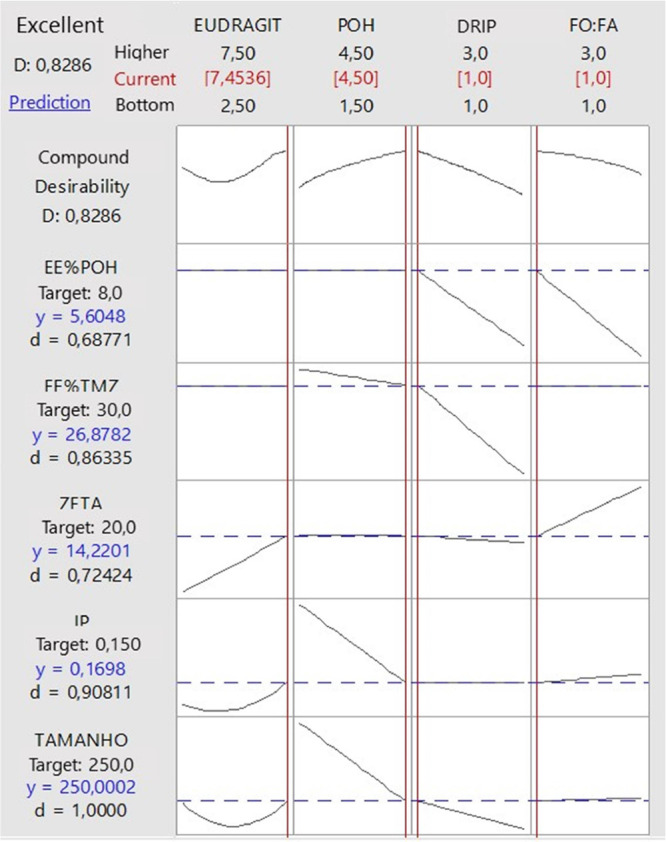
Chart of the response optimization result, achieving a desirability of 0.82.

Using the nanoprecipitation method, a composite desirability function approach was applied to identify the optimal experimental conditions for synthesizing TMZ+POH nanocapsules. This approach optimizes multiple response variables (*R*1, *R*2, *R*3, and *R*41/*R*42) by setting specific objectives. The aim was to minimize these response variables, with target values defined for each parameter, as detailed in Table 2. The optimization process and the corresponding outcomes are visually represented in Figure 12, clearly illustrating how the defined targets were achieved.

**Table 2:** Targets Defined for the Composite Desirability Test

parameter	goal	target
PS	target	200 nm
PDI	target	0.150
zeta potential	target	+20 mV
EE TMZ	target	20%
EE POH	target	8%

The desirability function, a dimensionless metric ranging from 0 to 1, evaluates experimental configurations to find the optimal balance of responses. A desirability value close to 1 indicates ideal conditions.^36^ The response optimization plot (Figure 12) revealed that the best configuration involved a high concentration of Eudragit (factor A), a high concentration of POH (factor B), a slow drip rate (factor C), and an equimolar O/W ratio (factor D). The composite desirability value approached 1, specifically reaching 0.8286, which indicates that the experimental setup closely aligns with the desired objectives for all response variables.

As shown in Figure 12, this method enables selection of conditions that optimize multiple responses simultaneously. Each column in the figure represents a process variable (Eudragit concentration, POH concentration, drip rate, and O/W ratio), while each row corresponds to a response or nanocapsule property (such as POH encapsulation efficiency, TMZ drug encapsulation efficiency, polydispersity index, and particle size). Solid lines indicate the trend of each response concerning changes in each variable, while dashed lines represent the target value for each response. Optimal parameters were derived by maximizing the overall desirability index (*D*), shown at the top of the figure. Each predicted value (*y*) and its desirability (*d*) relative to the target are listed beside each response, allowing for precise assessment of alignment with the set objectives.

Consequently, the optimal synthesis conditions for TMZ and POH nanocapsules via nanoprecipitation were determined to be a Eudragit RS100 concentration of 300 mg, an oil concentration of 180 mg, a drip rate of 1 mL·min^–1^, and an O/W ratio of 1:1 (v/v). Based on statistical analyses within the factorial design framework, these conditions will be adopted in subsequent research phases. This DoE approach enabled the assessment of interactive effects among factors, identifying optimal conditions to enhance the synthesis process of TMZ+POH nanocapsules via nanoprecipitation.

The mathematical models presented in this study demonstrate how certain parameters can be optimized to enhance the properties of the nanocapsule. Each equation derived for a specific variable influences the physicochemical characteristics of the nanocapsule, such as mean diameter, polydispersity index, zeta potential, and encapsulation efficiency.

When all variables are combined into a global response—calculated as the sum of each point divided by the highest value obtained for each parameter—the resulting model provides a broader and more generic perspective. However, this method does not indicate which variable is most affected but rather reflects the collective response of all parameters at a given point. Consequently, one aspect may improve while another might be compromised. Although this limitation may also occur in the separate analysis of variables, the latter approach offers a clearer and more detailed visualization, enabling targeted adjustments and a better understanding of how the nanocapsule responds.

This procedure allowed the identification of the optimal conditions necessary to optimize the process of obtaining TMZ+POH nanocapsules via the nanoprecipitation method.

### 3.2 Physicochemical Characterization of Eudragit Nanocapsules Containing TMZ and POH

Following the optimization of the production process for Nanocapsules containing TMZ and POH, physicochemical characterization was performed to evaluate the nanocapsule system using the nanoprecipitation method. The characterization involved various standard techniques to assess parameters such as average diameter, PDI, zeta potential, and morphology. Assessing these physicochemical properties is essential to understand nanocapsule interactions with biological membranes on both molecular and systemic levels.^37^

#### 3.2.1 Particle Size and Polydispersity Index

Particle size and size distribution are critical characteristics that influence factors such as biological fate, biodistribution, circulation time, toxicity, drug delivery efficiency, and the transport and release profiles of the therapeutic agent. Combined with zeta potential, these attributes determine the stability of the nanostructured system and its interaction with biological membranes.^27,38^

Table 3 presents values of 6 batches for average diameter (nm), PDI, zeta potential, and encapsulation efficiency for both loaded and unloaded nanocapsules, with measurements taken immediately after production to ensure stability. There was no statistically significant difference between empty and loaded nanocapsules. Maintaining a size below 300 nm is vital for intranasal administration effectiveness, as the nasal mucosa contains phagocytes that can capture foreign particles, making nanoscale formulations beneficial for overcoming this barrier.^17,39^

**Table 3:** Evaluation of the Particle size (PS), PDI and Zeta Potential of unloaded and loaded Nanocapsules^a^

formulation	PS (nm)	PDI	zeta potential (mV)	EE POH (%)	EE TMZ (%)
unloaded nanocapsules	251.4 ± 33.1^a^	0.164 ± 0.016^a^	19.50 ± 1.21^a^	N/A	N/A
TMZ+POH nanocapsules	252.6 ± 51.7^a^	0.145 ± 0.037^a^	20.98 ± 1.13^a^	3.66 ± 2.17	28.52 ± 5.95

a The results are expressed as the mean ± standard deviation (*n* = 6) from different lots of nanocapsules. There is no statistically significant difference between groups with identical letters (*p* < 0.05). N/A: non applicable.

**Figure 13:**
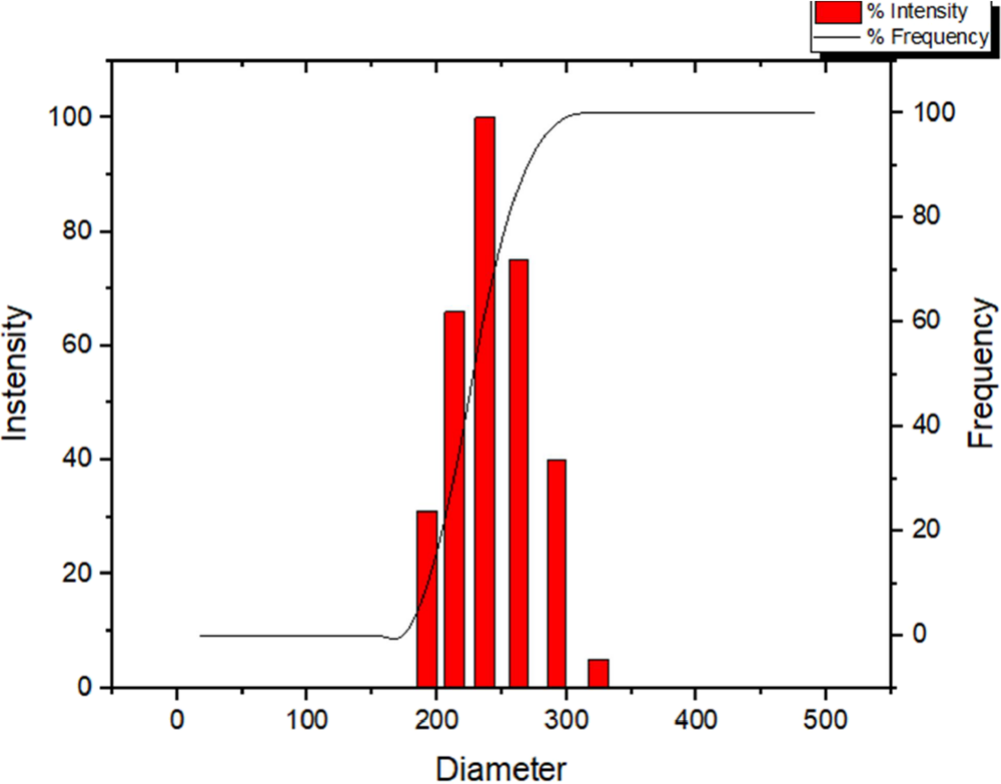
Size distribution of a representative sample of nanocapsules. Average diameter of 252 nm and IP of 0.145. monomodal profile.

Nanocapsules with diameters between 100 and 300 nm are particularly suitable for medical applications involving intranasal delivery. Such sizes reduce phagocytosis risk, enhance targeting efficiency through biological barriers, and enable controlled drug release due to the properties of the Eudragit RS100 polymer, which aids in drug retention. In vitro release tests are necessary to confirm the actual release profile 39–42. The mean diameters obtained in this study align with literature findings, with several studies using Eudragit RS100 reporting nanocapsule sizes in the range of 190–250 nm.^40−43^ Additionally, Eudragit RS100 enhances mucoadhesion^44,45^ a feature critical for intranasal drug absorption, allowing nanocapsules to remain in the nasal cavity and potentially traverse the olfactory and trigeminal nerves for targeted brain delivery.^9,45^Figure 13 illustrates the size distribution of the nanocapsules, showing an average diameter of 252 nm and a monomodal profile with a PDI of 0.145.

PDI measures the homogeneity of particle size distribution, with values ranging from 0.0 (perfectly homogeneous) to 1.0 (highly polydisperse with multiple distinct populations). Values ≤0.2 are generally preferred, indicating a more uniform distribution.^46,47^ The PDI of the TMZ and POH-loaded nanocapsules was 0.145 ± 0.037, demonstrating high uniformity in particle distribution and thus a stable formulation suitable for consistent delivery.

#### 3.2.2 Zeta Potential

Zeta potential is a key indicator of suspension stability in nanotechnology, as it relates to surface charge, which promotes electrostatic repulsion between particles, thus maintaining suspension stability.^31^ The average zeta potential was approximately +20 mV for both unloaded and loaded nanocapsules. Zeta potential values above or below 20 mV provide adequate electrostatic repulsion, preventing particle aggregation and maintaining Brownian motion, which is crucial for stable suspensions.^31,44^

Physiological pH (approximately 7.4) is a crucial factor for the stability and functionality of NCs, particularly concerning zeta potential. At physiological pH, the zeta potential directly influences the colloidal stability of NCs, as it determines the surface charge of the particles and, consequently, the repulsive forces between them.^31^ When the zeta potential is within a sufficiently high range (either positive or negative), greater electrostatic repulsion occurs, reducing the likelihood of particle aggregation and promoting suspension stability.^32^ In this study, the “optimal formulation” was identified based on a zeta potential that remained stable at physiological pH, ensuring uniform NC dispersion and controlled interaction with target cells. This is essential for in vivo applications, as inadequate stability could lead to NCs agglomeration and precipitation, compromising bioavailability and therapeutic efficacy.

#### 3.2.3 Nanoparticle Morphology

Morphology significantly affects the distribution, degradation, transport, and orientation of nanoparticles within biological systems, impacting cellular absorption, biocompatibility, and organ and tissue retention.^48^

**Figure 14:**
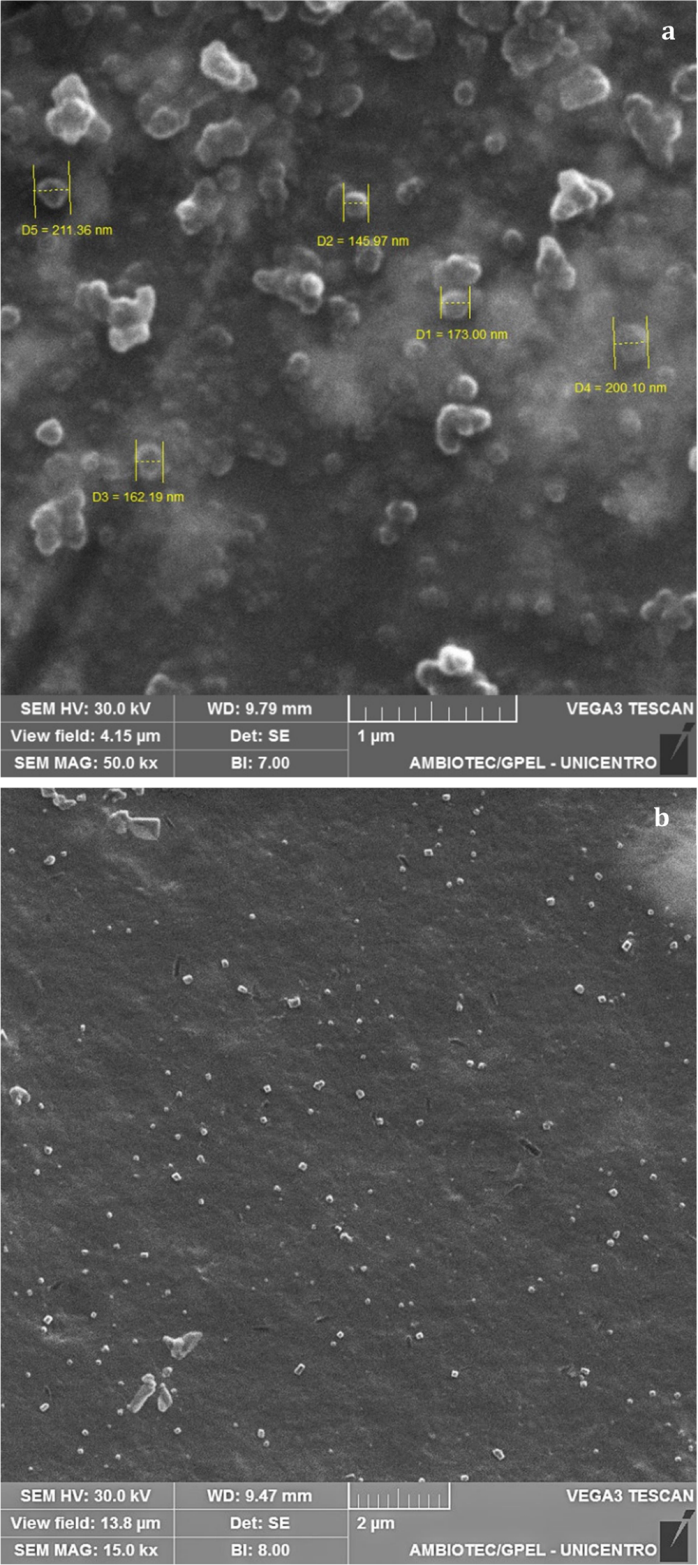
SEM images of TMZ+POH nanocapsules at 50,000× magnification (a) and 15,000× magnification (b).

SEM images (Figure 14) of loaded nanocapsules reveal a primarily spherical shape with sizes ranging from 146 to 212 nm, consistent with measurements obtained by DLS. This morphology aligns with findings from Santos et al., who reported similar spherical structures for Eudragit RS100 nanoparticles.^44^

Some degree of particle aggregation is visible in the SEM images. This aggregation is typical in nanoparticles and likely results from intermolecular interactions, such as van der Waals forces, which promote particle clustering in the dry conditions used for SEM sample preparation.^48^ However, these aggregates observed in SEM do not necessarily indicate aggregation in an aqueous suspension, as DLS measurements were conducted in liquid media, providing insights into nanocapsule behavior under conditions more representative of physiological environments.^32^

#### 3.2.4 Encapsulation Efficiency Percentage

The encapsulation efficiency for POH was low, at 3.66%, which may be due to Eudragit RS100s limited capacity to encapsulate and retain POH within the nanocapsule. This could be attributed to weak interactions between the polymer and POH. Conversely, TMZ showed a higher encapsulation efficiency of 28.53%, indicating a more favorable interaction with the polymer.

## 4 Conclusions

This study successfully applied a Design of Experiments (DoE) approach to optimize the preparation of Eudragit RS100 nanocapsules for coencapsulation of TMZ and POH using the nanoprecipitation method. The systematic evaluation of critical variables allowed for the identification of optimal conditions that produced nanocapsules with desirable physicochemical properties, including appropriate particle size, polydispersity index, and zeta potential. These parameters are essential for achieving stability and bioavailability in drug delivery systems, particularly for intranasal administration.

The optimized formulation achieved an average particle size under 300 nm, a PDI indicating homogeneity, and a stable zeta potential, all of which are favorable for intranasal delivery and enhance the potential for effective CNS targeting. Morphological analysis further confirmed a spherical structure, consistent with size measurements, while the system’s stability was validated through favorable zeta potential values, supporting its suitability for in vivo applications. However, despite optimization efforts, the encapsulation efficiency for POH remained low, potentially due to limited interactions with the polymer, while TMZ exhibited higher but still improvable encapsulation efficiency. These findings indicate the need for further refinement in the formulation process, potentially involving alternative polymers or encapsulation strategies, to enhance the encapsulation efficiency for both active agents. In summary, this research contributes to the development of nanocapsule-based delivery systems with promising characteristics for CNS-targeted therapies, specifically glioblastoma treatment.

## Supporting Information Available

The Supporting Information includes additional details regarding the experimental design and results. Table 1S presents the matrix of the full factorial design generated using Minitab software. Table 2S provides the results of the dependent variables used for optimizing the process of obtaining nanocapsules containing TMZ and POH via the nanoprecipitation method (PDF)
