# Supplementary material for: Optimization of Eudragit RS100 Nanocapsule Formulation for Encapsulating Perillyl Alcohol and Temozolomide Using Design of Experiments
Source: ACS Nanosci Au. 2025 Jan 29;5(2):70–83. doi: 10.1021/acsnanoscienceau.4c00057 (PMC12006857; doi:10.1021/acsnanoscienceau.4c00057)
Supplement: Supplementary file 1 — ng4c00057_si_001.pdf [file ng4c00057_si_001.pdf]

Optimization of Eudragit RS100 Nanocapsule Formulation for Encapsulating Perillyl Alcohol and Temozolomide Using Design of Experiments

Ariane K. P. Lorenzett[a]; Tatiane P. Babinski[a]; Vanderlei A. de Lima[b]; Rubiana M. Mainardes[a,c]*.

[a]Laboratory of Nanostructured Formulations, Universidade Estadual do Centro-Oeste, Élio Antonio Dalla Vecchia Aveniu, 838, 85040-167, Guarapuava - PR, Brazil

[b]Chemistry Department, Federal Technological University of Paraná UTFPR, Via do Conhecimento, s/n - KM 01 - Fraron, Pato Branco - PR, 85503-390, Pato Branco PR, Brazil.

*Pharmacy Department, Universidade Estadual do Centro-Oeste, Élio Antonio Dalla Vecchia Aveniu, 838, 85040-167, Guarapuava, PR, Brazil. *E-mail:* mainardes@unicentro.br

Supplemental Tables

Table 1S. Matrix of the full factorial design generated by the minitab software.

Formulation	X1	X2	X3	X4
**F1**	-1	-1	-1	1
**F2**	-1	-1	1	1
**F3**	0	0	0	0
**F4**	-1	1	-1	-1
**F5**	-1	1	1	-1
**F6**	1	1	-1	-1
**F3**	0	0	0	0
**F7**	1	-1	-1	-1
**F8**	1	-1	1	-1
**F3**	0	0	0	0
**F9**	1	1	-1	1
**F10**	1	-1	-1	1
**F11**	-1	-1	-1	-1

**F12**	1	1	1	1
**F13**	-1	1	-1	1
**F14**	1	-1	1	1
**F3**	0	0	0	0
**F15**	-1	-1	1	-1
**F3**	0	0	0	0
**F3**	0	0	0	0
**F16**	1	1	1	-1
**F17**	-1	1	1	1

Table 2S. Results of dependent variables for optimizing the process of obtaining nanocapsules containing TMZ and POH using the Nano-precipitation method. The independent variables or factors in this study are represented by the letters A (eudragit RS100 concentration). B (oil concentration - POH). C (drip) and D (ratio of organic phase to aqueous phase). In turn. the dependent variables. also known as response variables. are denoted by the letters R1 (mean diameter). R2 (polydispersity index). R3 (Zeta potential) and % EE of TMZ (R41) and POH (R42). Average±standard deviation.

FORMULATION	R1	R2	R3	R4[1]	R4[2]
**F1**	220.9±16.05	0.17±0.06	-0.96±7.81	33.15±1.95	0.68±0.85
**F2**	218.7±16.2	0.18±0.07	+4.45±2.82	42.52±3.91	4.24±3.65
**F3**	180.7± 9.7	0.16±0.03	+14.63±0.87	33.58±5.74	3.62±1.11
**F4**	255.3± 15.1	0.13±0.05	+7.96±4.18	21.53±9.77	5.24±0.89
**F5**	333.7±50.3	0.13±0.07	-0.69±1.47	11.52±2.93	0.35±0.23
**F6**	277.8±11.4	0.14±0.02	+11.90±1.3	34.24±2.50	1.62±0.37
**F3**	195.4±21.5	0.17±0.02	+17.47±4.41	35.25±19.87	3.51±2.77
**F7**	425.9±104.6	0.37±0.04	+15.03±1.42	46.73±4.60	10.52±3.49
**F8**	321.2±173.9	0.32±0.06	+14.83±9.42	23.62±4.47	0.55±0.26
**F3**	247.3±37.2	0.20±0.07	+8.95±1.27	38.73±20.78	4.01±1.23
**F9**	244.9±14.6	0.20±0.05	+18.30±5.14	22.53±12.54	1.35±0.34
**F10**	201.4±13.6	0.21±0.02	+16.83±1.3	27.36±3.36	0.33±0.28
**F11**	371.8±214.1	0.32±0.05	+10.13±3.46	7.15±1.17	3.56±1.48
**F12**	242.8±68.3	0.21±0.08	+18.53±2.83	25.68±2.83	0.66±0.23
**F13**	277.7±58.4	0.17±0.08	+2.55±2.08	26.02±4.03	1.46±1.46
**F14**	171.3±16.3	0.15±0.03	+26.40±7.91	56.23±10.40	6.22±3.04
**F3**	234.3±11.3	0.16±0.06	+15.87±1.89	32.66±1.44	3.99±0.50

**F15**	625.3±241.9	0.36±0.06	+21.13±3.46	37.69±24.12	2.63±0.11
**F3**	275.6±38.4	0.16±0.01	+9.02±2.23	25.43±14.20	4.69±0.88
**F3**	256.64±47.1	0.18±0.05	+17.731.19	22.84±10.90	5.62±3.61
**F16**	145.5±54.3	0.18±0.08	+18.80±1.35	15.66±1.35	2.592.63
**F17**	280.4±28.0	0.09±0.08	+1.50±1.17	28.55±7.24	4.15±1.92
